# Community-based pediatric palliative care: a systematic review of structures, processes, and outcomes

**DOI:** 10.1016/j.jped.2026.101510

**Published:** 2026-02-06

**Authors:** Sergio Andres Acuña-Caicedo, Aura Gisela González-Brossard, Laury Ellen Pérez-González, María Eugenia Arcia-Gálvez, Miguel Antonio Sánchez-Cárdenas

**Affiliations:** a Universidad El Bosque, Bogotá, Colombia; b Asociación Nacional de Enfermeras de Panamá, Ciudad de Panamá, Panamá

**Keywords:** Pediatric palliative care, Health services organization, Health process, Health Outcomes, Community Health Services, Pediatrics

## Abstract

**Objective:**

To analyze the structure, processes, and outcomes of different models of community-based palliative care of children, adolescents, and their families reported in the literature.

**Methods:**

The authors conducted a systematic review of original, full-text studies in MEDLINE and Embase (January 2014–May 2024), following PRISMA. Two reviewers screened/extracted data; a third resolved disagreements. Study quality and reporting were appraised with the Newcastle–Ottawa Scale and, as applicable, STROBE, COREQ, and CARE. Narrative synthesis was organized with Donabedian’s framework. PROSPERO: CRD42018747214.

**Results:**

Twenty-six high-quality studies (5 qualitative, 18 quantitative, 3 mixed-methods) described community PPC across home-based services, outpatient programs, community health and social support centers. Common structures included multidisciplinary teams with 24/7 availability, assistive devices/equipment, telemedicine, and screening/assessment tools (e.g., PedsQL, PedsQL-FIM, PIP, PaPaS). Core processes were home visits, caregiver education, symptom monitoring (including digital tools), advance care planning, interprofessional teamwork, and coordination with schools and social services. Reported outcomes most frequently included reduced hospital admissions and emergency use, improved symptom control and health-related quality of life, higher caregiver satisfaction, and lower parenting stress; however, measures and follow-up were heterogeneous.

**Conclusions:**

Community-based PPC can improve children’s and families’ quality of life when medical, psychosocial, and social resources are integrated beyond the hospital. To advance the field, validated outcome measures, sustainable financing, workforce training, and stronger policy frameworks are urgently needed.

## Introduction

Children, adolescents, their families, and health care systems face a significant challenge when advanced chronic diseases occur in childhood [1]. These children and adolescents experience a wide range of physical, emotional, and social symptoms that negatively impact their quality of life [2,3]. In this context, pediatric palliative care (PPC) is a critical tool for improving the quality of life of patients and their families. More than four million children and adolescents worldwide need palliative care, representing 7 % of the global need for palliative care [4]. However, this need is concentrated in regions such as Africa and Southeast Asia, where >70 % of children in need of palliative care live. HIV/AIDS remains the leading cause of palliative care in children, followed by premature birth and childbirth complications [4].

Traditional hospital-centric care often under-addresses psychosocial and spiritual needs and remains fragmented across services [3,5,6]. Community-based PPC reframes care around everyday contexts, home, school, and local services, linking medical, psychosocial, and social supports [7, 8, 9]. Depending on infrastructure and population needs, modalities typically include home-based programs, community health centers, social support centers/day programs, and compassionate-community approaches [7,9]. Using Donabedian’s structure, process, outcome lens allows clearer design and evaluation of these models [10].

In recent years, there has been a significant increase in care models that seek to adapt to the unique needs of each population. These care models demonstrate creative efforts to adapt PPC approaches to local needs, leveraging available resources and encouraging community participation [7,11,12]. The range of conditions requiring palliative care is broad and includes children with life-threatening health conditions where curative treatment is possible, children for whom premature death is inevitable, and children with progressive diseases for which curative treatment is not an option, requiring palliative care that may span years.

This research aims to analyze the structure, processes, and outcomes of community-based PPC models reported in the literature for the care of children, adolescents, and their families.

## Methods

A systematic literature review was conducted using the minimum standards described in the Preferred Reporting Items for Systematic Reviews and Meta-Analysis (PRISMA)[13] and the Palliative Care Literature Review Iterative Method (PALLETE) proposed by Zwakman et al. in 2018 [14]. This review complied with the approval protocols and was registered in the International Prospective Register of Systematic Reviews (PROSPERO) with the code CRD42018747214.

### Search strategy

A comprehensive literature search was conducted between January and May 2024 using the Medline (via PubMed) and Embase databases. Search strategies were developed using Medical Subject Headings (MeSH) for PubMed and Emtree terms for Embase, combined with relevant free-text keywords. Boolean operators and appropriate filters were applied to refine the results.

The specific search strategy used for PubMed was: (exp Child/ or exp Infant/ or exp Adolescent/ or "child*.mp.") AND (exp Palliative Care/ or "palliative care*.mp.") AND (exp Chronic Disease/ or "chronic illness*.mp.") AND (exp Models of Care/ or "models of care*.mp.") AND (exp Community Health Services/ or "community based*.mp." OR "home based*.mp.") AND (exp Quality of Life/ or "quality of life*.mp.") AND (exp Pain/ or exp Symptom/ or "symptom management*.mp.") AND (exp Family/ or exp Caregivers/ or "caregiver burden*.mp.").

The specific search strategy used for Embase was: (child OR infant OR adolescent) AND (palliative care OR "palliative care*") AND (chronic disease OR "chronic illness*") AND (models of care OR "models of care*") AND (community health services OR "community based*" OR "home based*") AND (quality of life OR "quality of life*") AND (pain OR symptom OR "symptom management*") AND (family OR caregiver OR "caregiver burden*").

The Rayyan® web-based platform was used to manage and screen the identified studies.

### Inclusion and exclusion criteria

Original articles in English published between January 1, 2014, and May 30, 2024, were considered eligible if they met the following criteria: (a) inclusion of conditions requiring palliative care according to the Association for Children's Palliative Care (ACT) classification, and (b) description of the structure, processes, or outcomes of community-based palliative care models, including home-based care services, community care centers for children and adolescents, day centers, compassionate communities, and hybrid models. The present study’s excluded articles that did not explicitly report that their population needed or received palliative care, non-original research articles (systematic reviews, meta-analyses, letters to the editor, short articles), and articles published in a language other than English or identified as duplicates in the initial literature search.

### Data extraction

Abstracts from the initial literature search were screened by two researchers for possible inclusion. The articles selected at this initial stage were read in full, and two researchers independently applied the inclusion and exclusion criteria of the review. A third researcher was consulted in case of discrepancies during the selection process. The reference lists of all selected articles were also screened to identify additional potentially eligible articles.

Three researchers on the team independently extracted data from the articles. Key information from each study was recorded in an Excel table created by the research group. The extracted data included study design, population, characteristics of the community intervention, and dimensions of quality (structure, processes, and outcomes). The extracted information was then reviewed by all researchers.

### Assessment of the risk of bias

The selected articles were classified according to their method as quantitative, qualitative, or mixed studies. The quality of the studies was assessed using different scales. The quality of experimental and cross-sectional studies was assessed using the Newcastle-Ottawa scale, which has nine items grouped into four sections: selection, comparability, outcome, and statistics. For each outcome of interest, validity scores were interpreted as follows: ≤ 5, low quality; 6–7, moderate quality; and 8–9, high quality. The STROBE statement was used to evaluate the quality of observational studies, the Consolidated Criteria for Reporting Qualitative Research (COREQ) to evaluate qualitative studies, and the Case Reporting (CARE) guideline to evaluate case series. Two reviewers independently assessed the quality of each study. A third researcher was consulted in case of disagreements during the quality assessment process.

### Data analysis and synthesis

The results were synthesized using Cochrane’s guidelines on synthesizing and presenting findings for systematic reviews of interventions [15], given the heterogeneity of studies evaluating various outcome measures; the main characteristics were synthesized narratively. The information extracted from the articles was reviewed and analyzed to identify common themes and representative conclusions. To better understand the results, the Donabedian model was used, which provides a classification of the dimensions of quality according to the structure, processes, and outcomes of health services. All authors participated in the analysis and interpretation of the results to ensure the integrity and accuracy of the process.

## Results

**Figure 1:**
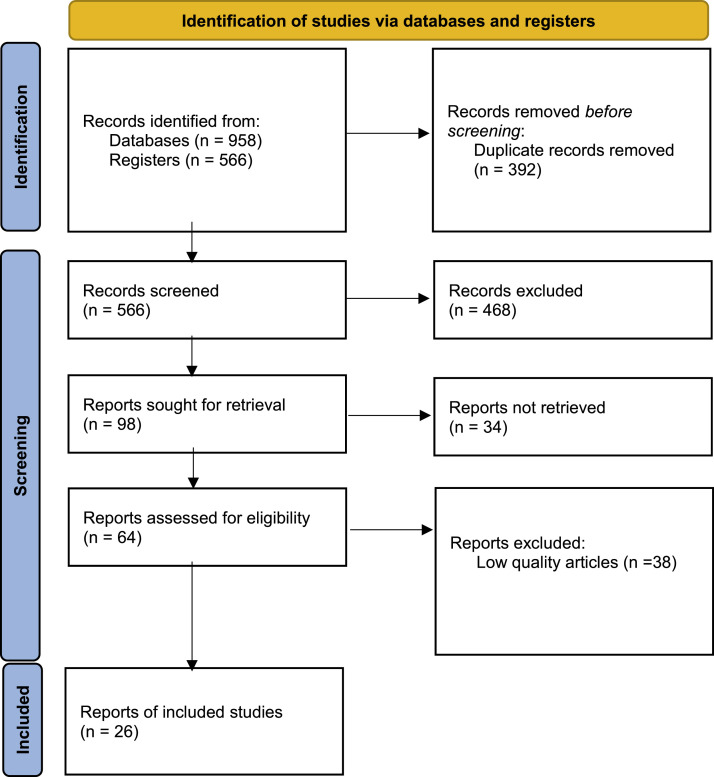
PRISMA flow diagram of the study.

A total of 958 articles were identified in the databases, of which 392 duplicates were removed. The initial screening focused on titles and abstracts of 566 studies and resulted in the exclusion of 468 articles. The full texts of 98 articles were then read to assess eligibility, resulting in the selection of 64 articles that met the inclusion criteria. These 64 articles were subjected to a quality assessment, and only 26 of them were classified as high quality. The latter 26 articles were finally included in the review (see Figure 1. PRISMA flow diagram).

The 26 studies on palliative care in children and adolescents were conducted in a variety of settings, including homes, community centers, hospitals, and outpatient units. Participants in the studies had multiple life-limiting health conditions, including cancer, neuromuscular disease, heart disease, chronic respiratory disease, congenital and genetic conditions, and HIV infection. From the selected studies, 5 qualitative studies (1 descriptive, 1 exploratory, and 2 thematic analysis studies), 18 quantitative studies (3 cohort studies, 5 clinical trials, 8 cross-sectional studies, and 2 observational studies), and 3 mixed studies were identified. Qualitative studies explored the palliative care experiences of children, adolescents, and their families, while quantitative studies examined palliative care interventions. The mixed studies used both qualitative and quantitative methods to gain a more complete understanding of child and adolescent palliative care and its quality dimensions (structure, process, and outcome) (Table 1) [16, 17, 18, 19, 20, 21, 22, 23, 24, 25, 26, 27, 28, 29, 30, 31, 32, 33, 34, 35, 36, 37, 38, 39, 40, 41].

**Table 1:** Summary of the studies included in the review.

**Lead Author**	**Method**	**Design**	**Setting**	**Conditions**	**Sample size**
Thienprayoon et al. [16]	Mixed methods	Quantitative and qualitative study	Home	Life-limiting conditions	63 members of the Ohio Pediatric Palliative Care and End-of-Life Network (OPPEN)
Vollenbroich et al. [17]	Quantitative	Cross-sectional study	Home	Inborn error of metabolism Congenital heart disease Oncological, neurological, cardiac, and other disorders	106 patients, 62 parents.
Peláez-Cantero et al. [18]	Quantitative	Cross-sectional study	Home Community hospital	Oncologic, neurologic, and neuromuscular processes.	944 patients
Visagie et al. [19]	Qualitative	Descriptive phenomenological study	Community center	HIV/AIDS infections	25 community health workers
Van Heerden et al. [20]	Qualitative	Exploratory study	Community center	Advanced cancer	39 stakeholder representatives
Ngwenya et al. [21]	Qualitative	Situational analysis study	Community center	HIV/AIDS infections	30 Health and social care professionals
Rost et al. [22]	Qualitative	Applied thematic analysis	Community center	Cancer	29 pediatric oncology providers
Grossoehme et al. [23]	Quantitative	Cohort study	Home	Genetic/chromosomal syndromes, neurologic or neurodegenerative conditions Cancer	209 patients
Jibb et al. [24]	Mixed methods	Quantitative and qualitative study	Outpatient units	Cancer	22 parents
Keefer et al. [25]	Quantitative	Cross-sectional study	Palliative care consultation	End-stage renal disease	35 patients
McCullough et al. [26]	Quantitative	Randomized clinical trial	Community health centers	Cancer	106 patients
Dussel et al. [27]	Quantitative	Randomized clinical trial	Outpatient cancer centers	Cancer	104 patients
Dalberg et al. [28]	Quantitative	Cross-sectional study	Cancer centers	Cancer	1005 pediatric oncology providers
La Fay et al. [29]	Quantitative	Cross-sectional study	Outpatient cardiologic centers	Cardiologic life-limiting condition	18 patients
Gallegos et al. [30]	Quantitative	Clinical trial	Pediatric oncology unit	Cancer	37 caregivers
Moen et al. [31]	Quantitative	Cross-sectional study	Community	Cerebral palsy	130 children and their families
Duberstein et al. [32]	Quantitative	Randomized clinical trial	Cancer care centers	Advanced cancer	30 caregivers
Ruskin et al. [33]	Mixed methods	Quantitative and qualitative study	Pediatric chronic pain program	Chronic pain	20 Patient and their caregivers
Din et al. [34]	Quantitative	Observational study	Home	Tracheostomized children	68 families
Tanner et al. [35]	Qualitative	Thematic analysis	Childhood cancer foundation	Cancer	58 patients 10 pediatric oncology workers
Simon et al. [36]	Quantitative	Observational study	Home	Cancer	27 participants 6 Healthcare professionals
Johaningsmeir et al. [37]	Quantitative	Cross-sectional study	Home	Children with medical complexity*	72 family caregivers
González et al. [38]	Quantitative	Cross-sectional study	Home	Neuromuscular disease, encephalopathy, central hypoventilation syndrome, apnea-hypopnea sleep syndrome, and chronic respiratory disease	167 patients and their families
Chong et al. [39]	Quantitative	Cohort study	Home	Neoplasms and non-cancer diseases	71 patients
Kuhlen et al. [40]	Quantitative	Cohort study	Home	Congenital malformations or chromosomal abnormalities	31 patients
Andriastuti et al. [41]	Quantitative	Randomized controlled trial	Home	Cancer	60 patients

⁎ > 5 specialists, > 3 organ systems or technologies and/or multiple medications.

### Characteristics of community-based pediatric palliative care models

Community-based PPC models cluster around four delivery settings—home-based care, outpatient PPC, community health centers, and social support centers—with cross-cutting strategies such as telemedicine, multidisciplinary teams, and educational tools[16,17,19,20,22, 23, 24,26,30,34,36, 37, 38, 39, 40, 41] Rather than repeating Table 2 [5,9,16, 17, 18, 19, 20, 21, 22, 23, 24, 25, 26, 27, 28, 29, 30, 31, 32, 33, 34, 35, 36, 37, 38, 39, 40, 41, 42, 43, 44, 45, 46], the authors highlight three consistent patterns:

(1) Structures that enable access (24/7 availability, equipment, assistive devices, digital monitoring) [26,28,38,39];
(2) Processes that integrate medical and psychosocial support (home visits, caregiver education, symptom surveillance, advance care planning, school and social-service coordination, interprofessional learning) [16,17,19,20,22, 23, 24,34,36,37,39,40]; and
(3) Outcomes that matter to families (fewer hospital encounters, better symptom control and HRQoL, higher satisfaction, and lower caregiver stress) [16,17,23,26,27,37,41].

**Table 2:** Characteristics of pediatric palliative care models by structure, processes, and outcomes.

**Modality**	**Structure**	**Process**	**Outcomes**
**Community health centers**	Community workers [16,23,24,36,38,39]	Health promotion and disease prevention activities[17,40]	Use of social resources[19,23,38,39,41]
		Psychological and psychiatric care [18,20,22,23,34,38,39,41,42]	Reduced incidence of preventable disease[34,36,38,40]
		Animal-assisted intervention[18]	Early integration of palliative care[24,34]
**Social support centers**	Social services[18,20,22,29,31,39,41,42]	Assistance with the child's daily living activities[17,20,24,34]	Social functioning[24,36]
	Assistive devices[17,20,25,34]	Approach to bereavement[32]	Bereavement control[32]
		Support groups[17,20,22,24,30,32,38,40]	Use of support services
		Integration of school resources[34,37,43]	Schooling[34,37,43]
		Psychological and psychiatric care[22,34,38]	Level of family distress[24]
**Outpatient palliative care**	Telemedicine teams[19,26]	Perception of symptoms by parents and healthcare professionals[19,23,39]	Family adherence[23,25,37,44]
	Mental health teams[5,9,24,27,32,36]	Psychological and psychiatric care[5,27,32,33]	Patient and caregiver preferences[5,23,29]
		Advance care planning[5,9,32,33]	Parenting stress reduction[18,19,24,39,41]
**Home-based palliative care**	Family caregiver availability[19,21,26,28,35,38,39,41,45]	Home visiting[19,21,26,28,35,38,39,41,45]	
	Assistive devices[19,25,28,37]	Assistance with the child's daily living activities[25]	Use of hospital resources[21,37]
	Supervision 24/7[21,28,39]	Transition management[17,22,24,36,38,40]	Parenting stress reduction[19,26,37]
	Clinical logistic system[38,39,45]		
**Cross-cutting modalities**	App-based solutions[19,26]		Symptom control[26,30]
	Multidisciplinary team[17,24,36,41]	Monitoring of symptoms and quality of life[19,26,28,37,44]	Health-related quality of life[28,37]
	Medical equipment and supplies[26,28,35,38,39]	Instrumental care[25,28]	Patient and caregiver satisfaction[18,27,28,39]
	Educational handout tools[17,29,34,36,38–40]	Counseling on symptom management[17,23,24,40,42]	Understanding care[18,19,24,27,29,32,39,46]
	Assessment instruments[17,22,31,34]	Educational interventions[19,21,22,26,29,31,32,38,39,41]	
	Clinical practice guidelines for pediatric palliative care[17,18,34,43]	Addressing psychological, social and spiritual aspects[17,24,34,35,38–40,42,43]	
		Care coordination[21,22,34,35,42,43]	
		Identification of palliative care needs[17,22,31,34–36,41,43]	

### Home-based care programs

According to the synthesized literature, the main structural components of these programs are related to the availability of a family caregiver [16,17,23,24,36,38, 39, 40, 41]. The studies consulted show that mothers [75.6 %] are the ones who usually devote themselves to caring for their sick child [38]. It is important to note that a multidisciplinary group of physicians, nurses, psychologists, social workers, and other specialists is needed to fully address the physical, psychological, and spiritual symptoms of children, adolescents, and their families [19,20,22,23]. It is essential to have 24/7 medical and specialized nursing supervision to ensure timely and adequate care, especially in crises, where the availability of technological equipment is crucial. Having this care available 24 h a day, 7 days a week, increases the likelihood of dying at home [18].

To meet the unique needs of children, adolescents, and their families, it is necessary to integrate rehabilitation teams, psychosocial care, and specialized nursing care [16,34,42]. According to the literature, using app-based solutions could improve synchronous monitoring and assessment of patient symptoms. The use of digital tools and mobile devices can facilitate communication, patient status monitoring, and remote support [24,36]. It is also important to note that assistive devices, such as wheelchairs, walkers, and patient lifts, can increase the mobility and autonomy of children and adolescents and improve their quality of life [24,31,34,38]. To ensure safe and effective home-based care, home programs must provide patients with all necessary medical equipment and supplies, such as hospital beds, infusion pumps, and medications, depending on healthcare system regulations [16,17,36,38,40]. The frequent use of a variety of assistive devices and the expected and perceived benefits demonstrate that early provision of assistive devices can be an effective strategy for improving the functional status of young children, especially in cases of severe functional dependency. The patient and family should receive educational materials to help them understand the disease [16,17,23, 24, 25,29,30,32,36,41], palliative care, and the home care process. An efficient clinical logistics system[16,17,39] is needed to transport professional staff and equipment, especially in rural or remote areas.

Family strain, including marital stress and reduced attention to siblings, was reported in multiple home-based PPC studies [17,38, 39, 40]. In these cohorts, qualitative data contextualized such percentages as reflections of high caregiver burden rather than generalizable population estimates. Table 1 summarizes each study’s setting, sample, and family-related findings to clarify these contextual differences.

According to the literature, it is critical that parents and healthcare professionals agree on how to assess the patient's symptoms to ensure that they receive appropriate care [5,18,30]. It is important to train family caregivers to manage the patient's symptoms at home. Equally important is ensuring that parents and healthcare professionals share a common understanding of symptoms to provide appropriate patient care [17,23,24,40]. Regular home visits by healthcare professionals should be made to monitor the patient's condition and adjust care as needed.

Palliative home visiting provides tailored care for patients and families, empowers parents to become skilled caregivers, and reduces 4.7 times unnecessary hospitalizations [41]. Children and adolescents should receive assistance with daily activities such as bathing, dressing, and eating; caregivers should also be provided with brief educational interventions on topics related to patient care [17,23,24,30,36,41].

Home-based care for children with palliative care needs should also address psychological, social, and spiritual aspects of patient care, with particular emphasis on providing psychological and psychiatric support for the patient and family [17,38,39]. Additionally, the literature emphasizes the importance of coordinating efforts among all healthcare providers to ensure comprehensive patient care [16].

Symptom control is one of the most important outcomes of palliative care. Several systematically reviewed articles describe patient and family caregiver satisfaction in terms of satisfaction with caregiving, though it is often emotionally overwhelming for families (65,9 %) [37,38]. The use of social resources is an important factor in PPC models. Among patients receiving home-based care, 71.8 % continued attending school. Studies also indicate that home-based preventive and follow-up care significantly reduces the use of hospital resources, including hospital admissions and emergency room visits [16,37,41]. Family outcomes also highlight the levels of family distress and understanding associated with home-based care. For patients who did not receive home visits, PPC teams often failed to provide comprehensive care, and parents did not have opportunities to discuss their preferences for the child's place of death, increasing the likelihood that the child would pass away in a hospital setting [18].

There is a pattern of late referral to community-based palliative care. In the study by Grossoehme et al. [23]. 27.2 % of patients died, and of these, 77 % were referred to home-based end-of-life care. This high percentage of deceased patients suggests that the notion of hospice versus palliative care may be a false dichotomy for many children with life-limiting conditions. In addition, the use of synchronous symptom assessment devices in these patients shows high rates of usability and acceptance by caregivers [36].

Several studies noted the needs of siblings of children receiving palliative care, who often experience disrupted routines, school difficulties, and emotional strain [17,38, 39, 40]. Programs that incorporated sibling support, such as psychoeducational sessions, recreational therapy, and school coordination, reported improved family functioning [17,24,39]. Likewise, models emphasizing interprofessional learning between nurses, physicians, psychologists, and educators showed enhanced care coordination, better symptom management, and greater caregiver confidence [19,20,23,36].

### Outpatient palliative care

Outpatient palliative care (OPC) services provide invaluable care and support to people with serious illness and their families. These services are characterized by a comprehensive, multidisciplinary approach that brings together a team of healthcare and social well-being professionals to provide individualized care [5,32,43]. It requires a structure with a clinical team of physicians and nurses to manage symptoms, prescribe medications, and coordinate care with other specialists [22,26,44]. The literature emphasizes the importance of involving rehabilitation teams, psychosocial care, and specialized nursing care [17,20,29]. Likewise, mental health services can play a crucial role in community palliative care. Including mental health professionals, such as psychologists and psychiatrists, and an interdisciplinary social support service can help individuals cope with the stress, anxiety, and depression associated with serious illness, as well as help patients and their families manage the social, financial, and legal aspects of the illness [18,25,27,32,33].

Using telemedicine improves access to palliative care, especially for patients living in remote areas or those with mobility difficulties [28,31,37]. OPC programs can offer online, in-person, or hybrid delivery models of PPC as telemedicine uses increase. While both patients and caregivers expressed a greater preference for in-person physical therapy sessions, they were more open to receiving psychology sessions online. Notably, 65 % of patients preferred in-person appointments, while caregivers expressed equal preference for in-person and online medical consultations [18,33].

The synthesized articles highlight the importance of OPC conducting a comprehensive assessment of the patient and family to understand their needs, priorities, and values [5,17,18,24,30]. Assessment of physical, psychosocial, and spiritual symptoms, as well as social and practical support needs, is included in OPC to effectively manage symptoms. Therefore, OPC should use a variety of techniques and include medications, complementary therapies, and non-pharmacological strategies. Advance care planning is highlighted as a key component of OPC because it allows families to express their wishes and preferences for how their children should be cared for during their final moments [5,9,32,45].

Coordination of care and education of patients and family members should be a central element of healthcare professionals, social services, and community organizations to ensure that the child, adolescent, and family receive comprehensive and consistent care that addresses all of their needs and ensures smooth transitions between different care settings, such as hospital, home, and school [16,23, 24, 25,29,30,32,35,36,41]. In addition, several of the studies underscore the need for bioethics consultation teams to manage ethical conflicts early in the care of end-of-life patients [22,28].

Some studies highlight the role of OPC in understanding pediatric palliative care as a resource for addressing children and adolescents' problems as well as the social functioning of patients and caregivers [20,21,44]. Reductions in hospitalizations and readmissions have been one of the most frequently reported management indicators in the literature, demonstrating the benefits of OPC [16,18]. Support for family members and caregivers in the bereavement process has been described, especially for patients with limited treatment options or when premature death is unavoidable. The quality and agreement of parents' and healthcare professionals' perceptions of symptoms influence quality of life and parental satisfaction and are predictors of quality of symptom management and palliative care [17].

### Community health centers

Community health centers should be equipped with structural resources to support comprehensive care, including a multidisciplinary team (e.g., psychologists, social workers, educators, and occupational therapists), individual and group consultation rooms, play and recreation areas, and a dining room stocked with didactic, recreational, and technological materials. These centers are required to provide a wide range of health services to children and adolescents, such as medical consultations, health screenings, immunizations, dental care, laboratory services, and nutritionist consultations [28,43, 44, 45]. In non-specialized community services, there is an important need for practical screening tools to identify the palliative care needs of children and adolescents and refer them to specialized services when necessary (e.g., the Pediatric Palliative Screening Scale (PaPaS)) [19,25,27,29].

Educational programs and materials for patients, families, health care professionals, and the community about PPC are the mechanisms to ensure adherence to treatment and connection to specialized services. This is particularly true for long-term chronic conditions and in health systems where early referrals to specialized PPC services are not yet standardized. The use of educational brochures has been shown to help caregivers become better informed and develop a greater preference for palliative care [30,32,43]. Healthcare professionals in these services should have access to up-to-date, evidence-based clinical guidelines for the care of children with serious or life-limiting illness. Additionally, validated tools need to be implemented to identify children who could benefit from palliative care promptly and effectively.

Given the frequent challenges faced by spouses with children receiving palliative care, community health centers should be able to provide support to families of children and adolescents regarding their physical, mental, and emotional health [17,26]. This may include psychosocial support and counseling, parenting and educational workshops, parent support groups, and connections to other social services [28,30,31,36,44].

Community health centers should promote health and disease prevention in children, adolescents and their families. Health and nutrition workshops, immunization campaigns, and sexual and reproductive health programs can be part of these promotion and prevention activities [5,43,45,46]. Family and social support, reduced parental stress, and reduced incidence of preventable illness in patients eligible for palliative care are common outcomes of this model of care [18,20,34,37].

### Social support centers

Social support centers (SSCs) for PPC patients and their families are intended to provide comprehensive support to children with chronic, complex, or terminal illnesses and their families [25,29,42]. Based on a biopsychosocial model of care, these centers aim to address the physical, emotional, social, and spiritual needs of patients and their families [5,25,26,41,45]. SSCs are typically staffed by physicians, nurses, psychologists, social workers, occupational therapists, educators, and support personnel. These centers are equipped with facilities, such as individual and group consultation rooms, play and entertainment areas for children, spaces for music and art therapy activities, libraries and reading rooms, as well as chapels or places for spiritual reflection [22,26,30,44].

According to the literature, SSCs offer a wide range of services and programs for patients and their families. These include symptom management, nutritional support, emotional assessment and counseling, individual and family therapy, support groups, and bereavement counseling [21,22,32]. Educational services for children with diseases requiring palliative care have developed significantly in these centers. These services include school support, coping skills workshops, health education, and play and recreational activities. Additionally, SSCS often provides resources such as religious or spiritual support and parent support groups [17,18,37].

Parents and families can also benefit from animal-assisted interventions [26], and it is possible to identify programs for patients with long-term chronic illnesses. SSCs have been shown to improve the quality of life for patients with chronic diseases and their families. Studies have shown that these centers can reduce patient and family stress and anxiety, improve mood and sleep quality, reduce caregiver burden, increase satisfaction with medical care, and ease the grieving process [32,33,36]. Improving the emotional and social well-being of children and adolescents, including reducing stress, anxiety, and depressive symptoms, and strengthening self-esteem and social skills, should be the primary outcomes of social programs [21,22,44]. Social support centers that provide care to children and adolescents with diseases that require palliative care should help them return to their community and school environments [16,47].

### Identified resources

The synthesis identified different resources essential for the care of children with conditions requiring palliative care, which are also integrated into different models of palliative care in the community setting (Table 3) [26,37,38].

**Table 3:** Resources identified in pediatric palliative care models by structure, processes, and outcomes.

Dimension	Characteristic	Resource	Description
STRUCTURE	Quality of life assessment	Pediatric Quality of Life Inventory 4.0 (PedsQL4.0)(38)	Tool to assess the quality of life of children and adolescents on home mechanical ventilation and their families.
	Impact of chronic disease on families	Pediatric Quality of Life Inventory Family Impact Module (PedsQL-FIM)(38)	Tool to assess the quality of life of children and adolescents on home mechanical ventilation and their families.
	Satisfaction with care	PedsQ™ Healthcare Satisfaction module(37)	Tool to assess parents' satisfaction with the overall care received by their children with medical complexity and high resource use.
PROCESS	Assessment of parental distress	Pediatric Inventory for Parents (PIP)(26)	Designed to measure stress in parents whose child has a chronic illness or requires prolonged medical monitoring.
	Assessment of impact of care		Study assessing the impact of caring for children with medical complexity and high resource use on families.
OUTCOME	Improvement of quality of live		
	Reducing the impact of chronic disease on families		
	Increased satisfaction with care	Healthcare Satisfaction module [PedsQL HCS](37)	It is expected that with the use of this tool, areas of satisfaction with care that need improvement can be identified.
	Improvement of the quality of life of children with cancer and their parents.		
	Improvement of the family well-being		

The studies included in the review reported some instruments useful for measuring the effectiveness of PPC interventions. One such tool is the Pediatric Quality of Life Inventory (PedsQL4.0), which was designed to assess structural parameters. This inventory can be administered to parents and even children, as it includes age-appropriate self-assessment questions addressing the child's condition in areas such as physical, emotional, social, and school functioning [26]. Another tool highlighted is the Pediatric Quality of Life Inventory Family Impact Module (PedsQL-FIM), a 36-item questionnaire that assesses the impact of chronic illness on families and the health-related quality of life (HRQOL) of children aged 2 to 18 years.(26) For process assessment, the Pediatric Parent Inventory (PIP) was reported. It measures the stress of primary caregivers of chronic pediatric patients using 42 items divided into 4 dimensions: medical care, communication, emotional distress, and role function.

## Discussion

This review shows that community-based PPC improves outcomes valued by families—symptom control, health-related quality of life, satisfaction—and can reduce acute care utilization when supported by accessible structures (e.g., 24/7 coverage, assistive devices, telemedicine) and robust processes (home visits, caregiver education, advance care planning, and interprofessional teamwork) [16,17,19,20,22, 23, 24,26,36, 37, 38, 39, 40].

However, evidence gaps persist: few randomized controlled trials, heterogeneous outcome measures, short follow-up, and limited reporting on economic and equity outcomes [18,34,39,41].

Policy and implementation lessons can be drawn from mature programs such as the UK’s community hospice movement and the Dutch Kindercomfort teams, which integrate PPC within primary care and education systems through stable financing, accredited training, and family-centered coordination [9,45,48]. Stronger alignment with WHO and AAP guidance — especially early integration, coordination of care, and inclusion of schools and social services — should guide model development [1,5,49].

Ethical and cultural dimensions remain insufficiently addressed. Early and iterative advance care planning helps align family values and clinical goals [5,9,28,32]. Programs that integrate culturally responsive communication and bereavement pathways support families more effectively and mitigate moral distress among professionals [17,35,38,39]. These aspects should become core components of community PPC training and implementation frameworks.

The review also highlights the difficulties of symptom management in PPC due to the limited availability of appropriate pediatric medications for children. In most cases, medication is prescribed without sufficient patient education, underscoring the need for research and development in this area. Likewise, the structure of home-based PPC programs is essential to ensure comprehensive, quality, patient-centered care [50]. Implementing the recommendations outlined in this review could strengthen the capacity of these programs to respond to the complex needs of children and their families. In addition, this review found evidence to support the effectiveness of several PPC interventions, including a nurse-coordinated palliative care approach. This strategy was found to be useful in addressing care needs across most PPC models. Caregivers who received educational resources about PPC reported less emotional stress during conversations about palliative care and when using digital technology [51]. Similarly, the use of digital devices in community care settings encouraged greater reflection, awareness, and communication [51].

This review also highlighted the need for specialized palliative care resources in community health centers. The availability of such resources would improve staff training and experience in caring for children with serious illnesses, even in hospice care settings. Although this review did not review studies of perinatal palliative care, it highlights the potential of these community-based programs to increase the availability and accessibility of care for these families [52].

Among the methodological limitations, although two widely recognized databases were used, it is possible that some relevant studies were not identified. However, comprehensive search strategies were applied in the grey literature to capture as many studies as possible. On the other hand, this review focused on literature published in English and Spanish, which may limit the inclusion of relevant studies published in other languages. Nevertheless, this decision aimed to ensure the quality and comprehension of the extracted data.

Finally, based on the resources identified in the literature, it is important to validate and assign pediatric indicators for evaluating PPC outcomes, conduct further research on the effectiveness of specific interventions in PPC, and develop and implement training programs for health professionals in community PPC. It is also essential to strengthen public policies that support community PPC.

## Conclusions

Community-based pediatric palliative care improves the quality of life of children and their families when care integrates medical, psychosocial, and social resources across home, school, and community. To strengthen evidence and scalability, four priorities emerge:

• Adoption of validated and standardized outcome measures (PedsQL 4.0, PedsQL-FIM, PIP, PaPaS) with longitudinal follow-up.
• Sustainable financing models that reimburse multidisciplinary community teams and assistive technologies.
• Workforce development through interprofessional training and supervision.
• National policy frameworks ensuring early integration, intersectoral coordination, and comprehensive bereavement support.

## Authors’ contributions

Sergio Andres Acuña-Caicedo: Designed the review protocol, screened articles following PRISMA guidelines, extracted data, constructed and synthesized the results, and drafted the manuscript.

Aura Gisela González-Brossard: Designed the review protocol, screened articles following PRISMA guidelines, and extracted data.

Laury Ellen Pérez-González: Designed the review protocol, screened articles following PRISMA guidelines, and extracted data.

María Eugenia Arcia-Gálvez: Designed the review protocol, screened articles following PRISMA guidelines, and extracted data.

Miguel Antonio Sánchez-Cárdenas: Designed the review protocol, screened articles following PRISMA guidelines, extracted data, constructed and synthesized the results, and drafted the manuscript.

All authors approved the final version of the manuscript.

## Data availability statement

The data that support the findings of this study are available from the corresponding author.

## Funding source

10.13039/100016018Universidad El Bosque.

## Conflicts of interest

The authors declare no conflicts of interest.
